# Posttraumatic stress disorder (PTSD) in the German Armed Forces: a retrospective study in inpatients of a German army hospital

**DOI:** 10.1007/s00406-012-0289-8

**Published:** 2012-01-25

**Authors:** Borwin Bandelow, Manuel Koch, Peter Zimmermann, Karl-Heinz Biesold, Dirk Wedekind, Peter Falkai

**Affiliations:** 1 Department of Psychiatry and Psychotherapy, University of Göttingen, von-Siebold-Str. 5, 37075 Göttingen, Germany; 2 Department of Psychiatry, Psychotherapy and Psychotraumatology, Bundeswehr Hospital, Berlin, Germany; 3 Department of Psychiatry, Psychotherapy and Psychotraumatology, Bundeswehr Hospital Hamburg, Hamburg, Germany

**Keywords:** Posttraumatic stress disorder, PTSD, Soldiers, Forces, Combat, Treatment

## Abstract

In 2006 and 2007, around 0.4 and 0.7% of all German soldiers involved in missions abroad were registered as suffering from PTSD. The frequency of PTSD in the German Armed Forces was assessed from army records. All soldiers admitted to the German Military Hospital in Hamburg, Germany, with PTSD (*n* = 117) in the years 2006 and 2007 were assessed by using questionnaires and structure interviews. Risk factors associated with PTSD were identified. Of the 117 soldiers with PTSD, 39.3% were in missions abroad, and 18.0% had participated in combat situations. Five (4.3%) were wounded in combat, and 4 of them had a serious irreversible injury. In total, 53.8% of the PTSD cases were related to injuries or physical/sexual abuse, while 46.2% were due to psychological traumatization. Among soldiers with PTSD who were not abroad, sexual or physical abuse were the most common traumas. In 35.9% of the patients, there was evidence for psychiatric disorders existing before the traumatic event. The percentage of women among sufferers from PTSD was significantly higher than the proportion of women in the armed forces (30.8% vs. 5.17%). A careful psychiatric screening before recruitment might help to identify persons at risk of PTSD.

## Introduction

Posttraumatic stress disorder (PTSD) develops in persons who experienced or witnessed events that involved actual or threatened death or injury or a threat to the physical integrity of self and others. There is also the possibility that persons who have killed, injured, or threatened other people may develop PTSD. Symptoms of PTSD include distressing recollections or dreams of the event, flashbacks, and stress at exposure to cues that resemble the trauma, diminished interest, insomnia, irritability, hypervigilance, depression, and anxiety. PTSD may occur with considerably delayed onset and may often become chronic. In an epidemiological study, 40% of subjects with PTSD still had the disorder after 6 years [36]. PTSD often co-occurs with mood, anxiety, somatoform, or substance abuse disorders.

The lifetime prevalence of PTSD was estimated to be about 6.8% in the US population [35]. Although a large number of people are involved in a threatening event, only a certain percentage develops PTSD. In a large random sample of American adults, 60.7% had been exposed to traumatic events; on average, 8.2% of men and 20.4% of women suffering from a comparable trauma develop PTSD [36]. The frequency of traumatic events may be different in the countries: In a large survey in Germany, 26% of male subjects and 17.7% of female subjects reported at least one traumatic event, but only 1% of males and 2.2% of females fulfilled PTSD criteria [52]. Varying prevalence rates were found for different forms of trauma; the proportion of individuals who developed PTSD was 53% for rape [36], 45.9% for kidnapping [24], 34.3% for being injured in a terrorist attack [51], and 9% for being involved in an accident [17]. A range of factors, other than the trauma itself, are needed to account fully for the pathogenesis of the disorder. Although men are more likely to experience trauma, the likelihood of developing PTSD is higher in women [9, 26, 64]. Subjects who develop PTSD have an increased ratio of psychiatric disorders before the trauma, such as preexisting affective, anxiety, or substance abuse disorders [1, 11, 43, 52]. Also, a family history of psychiatric disorders seems to be a predisposing factor [10, 13, 38]. Other risk factors include unstable family conditions during childhood [37] and sexual or physical abuse [8, 21, 23, 38, 50]. However, confounding factors that are correlated with childhood assault such as familial psychiatric disorders may be a possible bias. It was hypothesized that repeated traumatization may “sensitize” persons to the development of PTSD [21]. Persons who have been traumatized earlier have a higher chance to develop PTSD when they are re-traumatized. However, this seems to occur only when the earlier trauma also led to PTSD, according to a recent study by Breslau and Peterson [12]. Persons not developing PTSD after a prior trauma had no increased risk of PTSD after a subsequent trauma. According to the authors of this study, a “sensitization” hypothesis is not supported, but trauma may precipitate PTSD with preexisting susceptibility. Lack of social support after the traumatic event contributes significantly to the maintenance of PTSD symptoms [34]. Lower intelligence may increase the risk [46, 57]. Vulnerability or resilience to trauma stress may be determined by disruptions of neurotransmitter systems (e.g., serotonin, norepinephrine, glutamate, gamma-aminobutyric acid, substance P, neuropeptide Y and others) [58], or abnormalities of the hypothalamic–pituitary–adrenal axis were also observed [66]. Functional neuroimaging showed evidence that PTSD is associated with amygdala hyperactivity, hypoactivity in the medial prefrontal cortex, reduced hippocampal volume, and other abnormalities [18, 65]. There is a genetic disposition for developing PTSD [59]. Twin research to date suggests that exposure to trauma is moderately heritable, whereas exposure to non-assaultive trauma is not. PTSD symptoms are heritable, and comorbidity of PTSD with other disorders may be partly due to shared genetic and environmental influences [2, 39]. Environmental factors may interact with neurobiological changes. Childhood adversity was shown to be associated with later abnormalities in the HPA axis, such as persistent hyperactivity and chronic overactivity of CRF-containing neurons in limbic and hypothalamic brain regions [28].

PTSD is common among deployed servicemen of current wars. The prevalence of chronic PTSD was 15.2% among male American veterans who served in the Vietnam War [40], 12–13% in American veterans who served in Iraq and Afghanistan [30], and 3–6% in UK military personnel who deployed to the 2003 Iraq War [31]. Among US soldiers hospitalized following serious combat injury, 12.0% had PTSD 7 months after the trauma [27].

Some authors observed an increase in the prevalence of PTSD in the last wars [27, 47], while other authors do not see evidence for a rise over time, as the prevalence seemed to be higher in World War II and the Korean War than in recent wars [45]. The clinical presentation of PTSD has changed since World War I [41].

Risk factors for PTSD in soldiers have been in-vestigated. Higher educational level and higher cognitive ability were associated with a lower odds ratio for PTSD, privates had a higher risk than staff sergeants and officers, and combat soldiers had a higher risk than non-combat soldiers [68].

In general, PTSD is difficult to treat. Treatment modalities for PTSD include psychological and drug treatments, which have been evaluated by a number of expert committees, including the American Psychiatric Association [60], the UK National Institute of Clinical Excellence [49], the British Association for Psychopharmacology [3], the Australian National Center for PTSD [25], the US Institute of Medicine [32], the World Federation of Societies of Biological Psychiatry [4], and the Cape Town Consensus on Posttraumatic Stress Disorder [58]. Cognitive behavioral therapy (CBT), the most common psychological treatment for PTSD, was superior to a wait-list control or to a psychological placebo in most studies [4, 6, 49]. Findings on exposure therapy including confrontation with trauma-related stimuli were inconsistent, as positive results were found in some studies, but a deterioration was observed in others [55]. In order to prevent the development of PTSD, “debriefing”, a therapeutic conversation with an individual who has just experienced a traumatic event, was attempted. However, several studies even showed a worsening in the debriefing groups when compared to a control group [7, 22, 29, 44], while other studies did not show a difference [20, 54]. Meta-analyses of debriefing concluded that it does not improve natural recovery from trauma-related disorders [53, 61].

Eye movement desensitization and reprocessing therapy (EMDR) is being used widely for patients with PTSD. In an EMDR session, the client is instructed to focus on an image of a traumatic memory. Then, the therapist moves his fingers to the end of the patient’s field of vision, while the patient moves her/his eyes following the therapist’s fingers. Some therapists use sounds, tapping, or tactile stimulations. EMDR was more effective than wait-list/usual care control groups, according to a meta-analysis [6]. EMDR was shown to be effective in soldiers with PTSD in a non-randomized comparison with general hospital treatment and relaxation training [67].

Repetitive transcranial magnetic stimulation (rTMS) was effective in one controlled study [19].

According to a guideline on psychopharmacological treatment for PTSD [4], selective serotonin reuptake inhibitors (SSRIs) and serotonin norepinephrine reuptake inhibitors (SNRIs) are considered first-line treatments for PTSD. Second-line drugs are amitriptyline, imipramine, mirtazapine, risperidone, and lamotrigine, which have been shown to be effective in double-blind placebo-controlled trials. Prazosine may reduce nightmares. There is room for improvement in current pharmacological treatment strategies, as the response rates rarely exceed 60% [5].

After World War II, the German Armed Forces (Bundeswehr) were not involved in armed conflicts until 1992. Despite increasing engagement of the Bundeswehr in military conflicts, the numbers of injured soldiers and deaths in the German Armed Forces have decreased constantly (e.g., 1,176 deaths in the 1960s, 713 in the 1970s, 413 in the 1980s, 256 in the 1990s, and 222 from 2000 to 2008 [16], possibly due to increased safety standards in the armed forces. Ninety soldiers had lost their lives in missions abroad from 1992 to 2010.

In light of a 7.5-fold increase in the number of PTSD cases in the German Armed Forces from the year 2006 to 2010 (Table 1), it seemed necessary to investigate the prevalence of PTSD in the German Armed Forces and the characteristics and risk factors of the affected persons.

**Table 1:** Soldiers diagnosed with PTSD in the German Armed Forces in missions abroad in the years 2006–2010 [14, 15]

	Outpatients	Inpatients	All
2006	45	52	97
2007	94	68	162
2008	141	114	255
2009	279	200	479
2010	432	297	729

## Methods

The statistics for PTSD are given in Table 2. On average, 247,238 soldiers serve in the German army per year. Approximately 22,200 (around 11%) were stationed in combat areas in international missions in both 2006 and 2007; the exact numbers were not available from army sources. Of these, 97 (around 0.4%) and 162 (around 0.7%) were recorded as having PTSD in 2006 and 2007, respectively (Table 1).

**Table 2:** Traumatic experiences in soldiers of the German Armed Forces 2006–2007 [16]

	2006	2007	Total
Soldiers in the German Armed Forces 2006–2007	247,238	243,989	
Soldiers stationed in missions abroad (approximately^a^)	22,200	22,200	
All traumatic events in missions abroad	6,962	6,555	14,108
Soldiers injured or traumatized in missions abroad	5,630	5,130	10,740
Wounded in combat in missions abroad	13	17	30
Wounded by weapons or explosives	6	7	13
Death due to combat in missions abroad	1	3	4
Suicide in missions abroad	2	2	4
Soldiers with PTSD in the whole Bundeswehr	243	348	
Soldiers with PTSD who were in missions abroad	97	162	
Soldiers with PTSD admitted to German Military Hospitals	52	68	120
Soldiers with PTSD admitted to the German Military Hospital, Hamburg	62 (53.9%)	55 (38.2%)	117 (45.2%)

^a^Exact figures of soldiers stationed in missions abroad were not available from the German Armed Forces

Of all 243 soldiers stationed in Germany or abroad and diagnosed with PTSD in 2006 (348 in 2007), 115 were hospitalized in 2006 (144 in 2007) in one of the 4 German Military Hospitals (Berlin, Hamburg, Koblenz, Ulm). These included not only soldiers who were in combat areas, but all cases of PTSD in the army. All injuries of soldiers in missions abroad were registered; however, the available database does not allow differentiating the kind and severity of injuries.

The present study was conducted with all soldiers who had been treated for PTSD in the Hamburg German Military Hospital (total *n* = 117; 62 in 2006, 55 in 2007).

In Germany, drafting was mandatory until the end of February 2011. However, in 2006 and 2007, 187,931 (75.4%) of the soldiers were regulars. When recruited for the German army, soldiers are routinely screened using a psychological interview and have to undergo a group situation test, in which members of the Bundeswehr and psychologists examine candidates in interviews, watch them in role-playing and presentation exercises, and perform the Minnesota Multiphasic Personality Inventory (MMPI) and tests for intelligence, mathematics, and German language in order to exclude subjects with psychiatric disorders or intellectual disabilities from service. Although the German Armed Forces apply universal recruitment, all soldiers enrolled for service abroad (“out of area”) had to be regular soldiers and had to volunteer for this service. Missions of the Bundeswehr in the years 2006 and 2007 were Active Endeavour, Enduring Freedom, European Union Force (EUFOR), EUFOR Democratic Republic of Congo, International Security Assistance Force (ISAF), Kosovo Force (KFOR), United Nations Observer Mission in Georgia (UNOMIG), United Nations Mission in Ethiopia and Eritrea (UNMEE), United Nations Mission in the Sudan (UNMIS), United Nations Assistance Mission in Afghanistan (UNAMA), and Aceh Monitoring Mission (AMM). The areas of these missions included Afghanistan, Uzbekistan, Kosovo, Bosnia and Herzegovina, Congo, Sudan, Somalia, the Lebanon, and the Mediterranean Sea. Altogether, there is a contingent of 7,430 soldiers in these areas per year. The regular service is 17–26 weeks but lasts in fact between 4 and 26 weeks for every individual soldier, so that 22,200 persons were involved in these missions abroad (this number is only an approximate value, as the exact numbers could not be retrieved from Bundeswehr sources). There is no additional psychological screening for soldiers before serving in these areas.

The mean age of the 117 patients was 33.4 years (±SD 9.8) in the year 2006 and 31.8 years (±SD 8.7) in 2007. Thirty-six (30.8%) patients were women.

On admission, the following characteristics were recorded for all patients: age, gender, military rank, school education, number and duration of hospital admissions, operational missions and traumatic events such as injuries inside and outside army service, and sexual assaults. Combat-related events included also bomb assaults.

The diagnosis of PTSD was confirmed by experienced psychiatrists by a clinical interview and by using a checklist of PTSD symptoms according to the Posttraumatic Stress Scale (PTSS-10 [62]) in the German translation [42] and the Impact of Event Scale—Revised (IES-R; [63]).

Patients were also screened for other psychiatric disorders, including major depression, panic disorder/agoraphobia, generalized anxiety disorder, social anxiety disorder, somatoform disorders, personality disorders, eating disorders, and substance abuse. The history of the patients was screened for the evidence for psychiatric problems existing before army service by a thorough analysis of the Bundeswehr file, the recruiting file, and available physicians’ charts.

Patients underwent routine medical examination including laboratory tests. All psychopharmacological and other medications prescribed by the hospital physicians during admission were recorded.

Psychological treatment modalities offered to the patients included cognitive behavioral therapy (CBT), eye movement desensitization and reprocessing therapy (EMDR) [56], and “psychological debriefing” directly after their trauma exposition.

At discharge of the hospital, success of treatment was rated by a simple yes/no statement by the treating physician after a retrospective analysis of the charts. Also, ability to work was recorded (fit for Bundeswehr service/fit for any work/retirement plan) by the assessment of a professional surveyor who was not identical with the treating therapist.

Patients gave written consent to the storage and use of their medical charts. The retrospective evaluation was approved by the institutional ethical board of the University Hospital of Göttingen, Germany.

## Results

Table 2 summarizes the statistics of PTSD in all German soldiers in the years 2006–2007. In the table, all kinds of injuries are listed, irrespective of severity degree. From the available database, it was not possible to differentiate between mild and severe injuries. Of all soldiers, 31.5 and 31.1% stationed in missions abroad were injured in 2006 and 2007 (this includes all severity grades from mild to severe).

Around 0.4 and 0.7% of all soldiers stationed in combat areas were registered as having PTSD in 2006 or 2007, respectively (as the number of soldiers stationed in missions abroad 22,200 is only an approximate value, these percentages may not be exact).

In Table 3, the traumatic experiences of the soldiers are summarized. Of the 117 soldiers with PTSD, 46 (39.3%) were in missions abroad (28 in 2006 and 18 in 2007). Thus, our sample comprises 53.9% of all PTSD inpatients of the German Armed Forces who were in missions abroad in 2006 and 26.5% of all PTSD inpatients in 2007.

**Table 3:** Traumatic experiences in 117 soldiers of the German military hospitalized in the German Military Hospital, Hamburg, in 2006–2007

	*N* (%)
PTSD patients who were in missions abroad	46 (39.3%)
Injury in combat (forefoot amputation acoustic trauma upper ankle joint distortion splinter/blast injury bruise)	5 (4.3%)
Psychological traumatization only	41 (35.0%)
Being in combat without injury and being witness of severe injuries or death of other persons in combat	16 (13.7%)
Witness of severe injuries or death of other persons in combat	16 (13.7%)
Psychological traumatization by assault with improvised explosive device (IED)	4 (3.4%)
Psychological traumatization by assault by civilians without military weapons	5 (4.3%)
PTSD patients who were not in mission abroad	71 (60.7%)
Injury/abuse	58 (56.4%)
Traffic accident (1st-degree cranial trauma, broken leg; complicated fracture of tibia)	8 (13.7%)
Almost drowned	1 (0.9%)
Physical abuse during childhood/adolescence, within the family	13 (11.1%)
Physical abuse during childhood/adolescence, other	13 (11.1%)
Sexual abuse, including abuse during childhood/adolescence	23 (19.7%)
Psychological traumatization only	13 (4.3%)
Being in a traffic accident	8 (13.7%)
Witness of severe injuries or death of other persons	5 (4.3%)
All PTSD patients	117
Injury/abuse	63 (53.8%)
Psychological traumatization only (e.g., witness of severe injuries or death of other persons/life-threatening event)	54 (46.2%)

Of the soldiers in missions abroad, 21 (18.0%) had participated in battle situations. Five (4.3%) were wounded in combat, and 4 of them had serious irreversible injury (forefoot amputation, blast injury, complicated fracture of tibia, acoustic trauma). The remaining soldiers suffered from psychological traumatization (e.g., being in combat or in an explosives assault without being injured and being witness of severe injuries or death).

Among the 71 (60.7%) PTSD patients who were not in missions abroad, 58 (56.4%) were injured, mostly in traffic accidents, or had traumatization by physical or sexual assault. Thirteen (4.3%) had exclusively psychological traumatization.

In total, 64 (53.8%) of all PTSD cases were related to injuries or physical/sexual abuse, while 54 (46.2%) were due to only psychological traumatization.

### Comorbid disorders

Comorbid disorders existing before the traumatic event included major depression (*n* = 33; 28.2%), panic disorder/agoraphobia (*n* = 8; 6.8%), social anxiety disorder (*n* = 1; 0.9%), somatoform disorder (*n* = 4; 3.4%), emotionally instable personality disorder (*n* = 5; 4.3%), and eating disorders (*n* = 8; 6.8%), including 3 with binge eating disorder (2.6%), 1 with obesity (0.9%), 2 with bulimia nervosa (1.7%), and 2 with anorexia nervosa (1.7%). Nicotine abuse in 24 (20.5%) cases and cannabis abuse in 3 cases (2.6%) were reported. Altogether, in 35.9% of the patients, there was evidence for psychiatric problems existing before the traumatic event, according to the available documents.

Comorbid conditions that emerged after the traumatic events included alcohol abuse (*n* = 7; 6%), alcohol addiction (*n* = 3; 2.6%), and cocaine abuse (*n* = 1; 0.9%).

### Risk factors

The percentage of women among sufferers from PTSD was significantly higher than the proportion of women in the army (30.8% vs. 5.17%; *P* < 0.0001). This percentage was above the expected score (χ² = 161.4; *df* = 1; *P* < 0.0001), as only 5.17% of all soldiers serving in the army in the years 2006–2007 of the German Armed Forces were women [16].

A high number of women (*n* = 23; 19.7%) reported to have been sexually traumatized. In 21 cases, this happened before the Bundeswehr service, while in 2 cases, the attack occurred during the army period. However, in both of these cases, the women were not assaulted by Bundeswehr soldiers, but at home in their family surroundings.

The frequency distribution of military ranks was compared with the distribution in the whole army (Table 4). The percentage of non-commissioned officers was significantly higher, and the percentage of privates was lower than in the whole armed forces (Χ^2^ = 16.9, *df* = 2, *P* = 0.0002), whereas the percentage of officers was not higher than expected.

**Table 4:** Military ranks, comparison of PTSD sample with frequency in the whole Bundeswehr

	Privates	Non-commissioned officers	Commissioned officers	Total
PTSD sample	23 (19.6%)	76 (65.0%)	18 (15.4%)	117
German Armed Forces	91,865 (37.1%)	117,939 (47.7%)	37,434 (15.1%)	247,238

School education in the PTSD sample differed from the distribution in the whole German Armed Forces population. Secondary school graduates were significantly overrepresented in the PTSD sample, whereas primary and high school graduates were less frequent than in the total Bundeswehr (Χ^2^ = 9.5, *df* = 2, *P* = 0.008) (Table 5).

**Table 5:** Comparison of the school education of the sample with the distribution in the whole army (average from the years 2006 and 2007)

	Primary school	Secondary school	High school	Total
PTSD sample	30 (17.9%)	66 (56.4%)	21 (25.6%)	117
German Armed Forces	54,089 (22.0%)	114,959 (46.8%)	76,541 (31.2%)	247,238

### Treatment

The total number of impatient treatment stays of the 117 patients was 264, corresponding to an average of 2.3 treatments per patient (range 1–22 treatments). The average treatment duration was 47.7 days. PTSD patients treated in 2006 and 2007 had a total of 5,590 days of treatment. As the treatment costs in the hospital are 231 Euro a day, treatment costs summed up to 1,291,290 Euro for the total sample and 11,036 Euro per patient.

All 117 patients have been treated with psychological treatment interventions. These included cognitive behavioral therapy (CBT) (*n* = 15; 12.8%) and eye movement desensitization and reprocessing therapy (EMDR) (*n* = 102; 87.2%).

Twenty-two (18.8%) of the soldiers were treated with psychopharmacological drugs. In 13.7% of all patients, antidepressants were used (SSRI, 4.3%; SNRI, 0.9%; TCAs, 2.6%; mirtazapine, 6.0%). Benzodiazepines and zolpidem were used in 5.2% and low-potency antipsychotics in 0.9%. A total of 8.5% of the patients received 2 or more psychopharmacological drugs. In 22 cases (18.8%), patients received a dose below the normal range, and in 16 cases (13.7%), drug doses were not titrated up to the maximum allowed dose according to the package insert.

### Outcome

The complete treatment setting, including EMDR, CBT, medication, and other supportive strategies, was considered successful in 91 (77.8%) of the patients, according to the assessment of the treating therapists, while 22.2% were not considered as markedly improved. In 91 (77.8%) of the 117 cases, the patients reported that the psychological treatment had been successful, whereas in 81.3% of the 22 cases, drug treatment had been rated as successful.

Ninety-one (77.8%) of the patients were fit for service again after treatment, while 3 (2.6%) were fit with limitations, and 23 (19.6%) were unfit for work. Three (2.6%) of the patients had a degree of restricted ability to work of 25% or more, according to the assessment of professional surveyors.

## Discussion

Around 0.44 and 1.15%, respectively, of the soldiers stationed in combat areas in the years 2006 and 2007 developed PTSD. This percentage is lower than that in previous studies which found percentages of 3–15.2% in combat soldiers. However, the risk of developing PTSD is dependent on the severity of combat activities, and the role of the German Armed Forces in the missions abroad in 2006 and 2007 was more the one of a peace-keeping force. In these years, combat situations and assaults with explosives were less frequent than today. It may be assumed that the percentage of PTSD has increased since then and will further increase, as the armed conflicts in Afghanistan and other countries, in which the Bundeswehr is stationed, have increased in number. For example, the death toll of the Bundeswehr in missions abroad was one soldier in 2006 and 3 in 2007, while in 2010 and 2011, 9 and 7 soldiers, respectively, were killed in Afghanistan [16]. There was a 7.5-fold increase in the number of PTSD cases in the German Armed Forces from the year 2006 to 2010. Thus, it is likely that PTSD in German soldiers may be increasingly important in the future.

A total of 46 (39.3%) of the PTSD cases were soldiers who had been in missions abroad. In only five cases, PTSD was associated with injury in combat. In the majority of the remaining cases, the affected persons did not experience actual damage to the physical integrity of their self but were exclusively traumatized psychologically, for example by being in combat or in an explosives assault without being injured, and being witness of severe injuries or death of other persons. Our sample comprises 53.9% of all PTSD inpatients of the German Armed Forces in 2006 and 26.5% of all PTSD inpatients in 2007. Therefore, it may be seen as fairly representative for the whole group of PTSD inpatients from the German Armed Forces. The relatively low number of severely injured persons in our sample has to be seen in the context that in the whole German Armed Forces, approximately one third of soldiers in missions abroad were wounded at least once. However, the available database does not allow differentiating between the different degrees of severity of these injuries. Thus, also minor injuries were included. Nevertheless, it is likely that the majority of soldiers who were injured did not develop PTSD.

Among the non-combat-related events associated with PTSD, sexual and physical abuse was most common.

There is the possibility that PTSD symptoms are malingered or exaggerated by war veterans in order to apply for financial compensation [45]. On the other hand, there may also be a substantial number of unreported cases, for example when a soldier does not want to lose her or his army employment or does not want to be seen as a “coward” or “weakling” by his close relatives or friends, when complaining about distressing PTSD symptoms. The stigma of psychiatric illness may also be a reason for underreporting of PTSD symptoms. In order to determine the actual prevalence of PTSD in soldiers and veterans, an anonymous survey would be necessary. To our knowledge, such investigations are underway.

The frequency of various symptoms differed between non-combat- and combat-related PTSD. Nightmares and suicide attempts were the most common symptom in non-combat-related PTDS, while intrusions or detachment/estrangement from others was most common in combat-related PTDS.

Among the comorbid psychiatric conditions, major depression and anxiety disorders were most common. PTSD can be more easily triggered in persons who had pre-existing psychiatric disorders, such as anxiety disorders and depression. Likewise, in our sample, in approximately one-third of the patients, preexisting psychiatric disorders were found, thus supporting a theory of proneness to PTSD as a potential risk factor.

All cases of alcohol abuse or addiction developed after the trauma, according to the reports of the patients in our sample. Alcohol abuse is common in patients with PTSD [33].

Women were significantly overrepresented in the PTSD group. Given the high number of sexual assaults, all of which did not happen within army settings or under war conditions, the gender specificity may partly be explained by an increased risk of sexual traumatization in women. However, it is not possible to determine whether early sexual assault significantly increased the risk of PTSD in our sample, as a comparison with an age-matched sample of non-army women is lacking. The available evidence from other studies suggests that the sex difference is not due the higher occurrence of sexual assault among females, prior traumatic experiences, preexisting depression or anxiety disorder, or sex-related bias in reporting [9, 26].

The percentage of non-commissioned officers was significantly higher, and the percentage of privates was lower than in the whole Bundeswehr, whereas the percentage of officers was not higher than expected. This is in contrast to other investigations, in which privates had a higher risk than staff sergeants and officers [68]. Secondary school graduates were more frequent in the clinical sample, whereas high school and primary school graduates were a little less frequent than in the whole Bundeswehr. This is partly in contrast to other investigations, in which higher education level and higher cognitive ability were associated with a lower odds ratio for PTSD [68]. We do not have a plausible explanation for this finding of higher PTBS rates in middle-ranked soldiers or in probands with medium-level education.

In the present study, PTSD treatment followed a naturalistic setting. EMDR was used in the majority of patients (87.2%). Debriefing, which nowadays is seen as an obsolete method because it even can increase the risk of the development of PTSD in traumatized persons, was applied in two cases directly after an impact in Afghanistan. Drug therapy was only applied in 18.8% of the patients. This is in contrast to other investigations with traumatized soldiers. For example, 80% of PTSD patients treated by the US Veteran’s Administration received psychotropic medication, mostly with antidepressants and less frequently with sedative drugs or antipsychotics [48]. Some patients in the present sample did not receive drugs that were shown to be effective in PTSD [4], and doses were too low or were not uptitrated the maximum recommended doses, which is suggested for unresponsive cases of PTSD.

In all armed conflicts, the possibility of the development of PTSD has to be taken into account. For the future drafting of soldiers, it may be recommended to apply a carefully structured predrafting psychological assessment, although such predrafting failed to identify possible risk factors for PTSD [68]. Persons trying to apply for a position in the army may not fully disclose pre-existing psychological problems in order not to fail in the drafting procedure.

When women are drafted for the army, one has to take into account that this will result in a higher rate of PTSD. Persons who have been traumatized earlier may have an increased risk of developing PTSD.

### Limitations

Not all German Armed Forces soldiers suffering from PTSD in 2006 and 2007 were registered in this study. This sample included only 17.8% of all reported PTSD cases in the German Armed Forces, but 45.2% of all inpatient PTSD cases admitted to German Military Hospitals. Therefore, the study results may not be representative for the whole group of PTSD patients but may be characteristic for the more severe inpatient cases of PTSD.

It is difficult to draw conclusions from the treatment outcome data, which were gathered in a naturalistic setting. As this was an open, retrospective analysis, it is not clear whether improvement was due to the applied psychotherapeutic methods, drug therapy, unspecific attention effects, or spontaneous remission. Assessment of improvement was only done by global impression, without the use of standard PTSD scales. Although a high proportion of patients were considered fit for service again after treatment, information could not be gathered from the available files whether the individuals did in fact return to active duty. Further studies should use a randomized, prospective design and should apply established rating scales.
